# Sperm freezability is associated with melatonin receptor expression in the sperm plasma membrane of Saanen goat bucks *(Capra hircus*)

**DOI:** 10.14202/vetworld.2025.3520-3535

**Published:** 2025-11-27

**Authors:** Alberto J. Cardenas-Padilla, Alfredo Medrano

**Affiliations:** Secretaría de Posgrado e Investigación, Unidad de Investigación Multidisciplinaria, Laboratorio de Reproducción Animal (L2), Facultad de Estudios Superiores Cuautitlán, Universidad Nacional Autónoma de México

**Keywords:** cryopreservation, freezability, goats, melatonin receptors, seminal plasma, spermatozoa

## Abstract

**Background and Aim:**

Cryopreservation is an essential assisted reproductive technology for preserving valuable animal genetics; however, it induces osmotic and oxidative stress that compromises sperm quality. Melatonin (MLT) functions as both an antioxidant and a signaling molecule through specific membrane receptors, melatonin 1 (MT1) and melatonin 2 (MT2). This study aimed to determine the association between sperm freezability, MLT receptor expression on spermatozoa, and MLT concentration in the seminal plasma of goat bucks.

**Materials and Methods:**

Semen samples were collected weekly from seven clinically healthy Saanen bucks (2–3 years) maintained under natural photoperiod and standardized feeding conditions. A total of 124 ejaculates were analyzed seasonally (spring-winter). Sperm quality parameters, including motility, viability, capacitation status (chlortetracycline [CTC] assay), plasma membrane fluidity, and acrosome integrity, were evaluated before and after cryopreservation. Expression levels of MT1 and MT2 receptors were quantified by Western blot, while MLT concentration in seminal plasma was measured by enzyme-linked immunosorbent assay. Pearson’s correlation and determination coefficients (R, R^2^) were computed to assess associations between sperm quality, receptor expression, and MLT concentration.

**Results:**

Western blot analysis revealed variable MT1 (16 kDa) and MT2 (28–75 kDa) expression throughout the year. High negative correlations (R > −0.9, R^2^ > 0.8) were observed between the expression of both receptors and the proportion of acrosome-reacted (AR) spermatozoa (CTC-AR pattern), except for MT2 (75 kDa). Conversely, seminal plasma MLT concentration showed a strong positive correlation (R > 0.9, R^2^ > 0.8) with capacitated sperm having intact acrosomes (CTC-B pattern). Seasonal variation in receptor expression, rather than MLT concentration, influenced sperm cryoresistance.

**Conclusion:**

Sperm freezability in goats is closely linked to the expression of MLT receptors and seminal plasma MLT concentration. Higher receptor expression corresponds to improved post-thaw sperm quality, likely by mitigating cryocapacitation and acrosomal damage. These findings suggest that enhancing MLT receptor expression or modulating photoperiodic exposure could optimize semen cryopreservation protocols and reproductive efficiency in caprine species.

## INTRODUCTION

Melatonin (MLT) (N-acetyl-5-methoxytryptamine) is an amphiphilic indolamine synthesized from the amino acid tryptophan [1, 2]. In vertebrates, the pineal gland serves as the principal site of MLT synthesis and secretion, primarily during the scotophase of the circadian rhythm [3]. Beyond regulating the biological clock and sleep-wake cycle, MLT plays a critical role in modulating reproductive functions in seasonal breeders [4]. In short-day breeders such as goats (*Capra hircus*), MLT acts as a key endocrine signal stimulating reproductive activity [5]. Consequently, variations in sexual behavior and semen quality are observed throughout the year, with marked declines during the non-breeding season [6].

In addition to its endocrine function, MLT exerts potent antioxidant effects, acting as a free radical scavenger at both systemic and local levels [7]. Its antioxidant capacity is mediated not only by the parent molecule but also by its metabolites. Metabolomic studies have identified approximately 14 MLT-derived compounds. MLT is converted to kynuramine derivatives, including indoleamine-2,3-dioxygenase and N^1^-acetyl-N^2^-formyl-5-methoxykynuramine (AFMK), which can subsequently transform into a more stable metabolite, N^1^-acetyl-5-methoxykynuramine (AMK). AFMK and AMK are among the most potent MLT metabolites with antioxidant activity [8]. Locally, MLT enhances spermatogenesis and steroidogenesis, improving testicular and seminal parameters [9, 10]. Males with higher endogenous or supplemented exogenous MLT levels typically demonstrate increased libido and superior seminal quality [11].

Several MLT effects are mediated through specific membrane and nuclear receptors. Two well-characterized plasma membrane receptors, MT1 and MT2, both G protein-coupled receptors with seven transmembrane domains, have been extensively identified in mammalian reproductive tissues [12–18]. These receptors are expressed in the ovaries and testes [19] and have been localized to the sperm plasma membrane across multiple species, including humans, rams, donkeys, stallions, boars, bulls, dogs, camels, and goats [20–24]. Activation of these receptors triggers signaling cascades associated with plasma membrane reorganization and receptor internalization [25–27]. Such desensitization mechanisms have been demonstrated for MT1 in humans, mice, and rams following transient MLT exposure [28, 29]. In goats, the distribution of MT1 and MT2 receptors suggests their participation in sperm capacitation and acrosome reaction [24]. Although the specific signaling mechanisms in goats remain to be elucidated, studies in other species indicate that MLT inhibits epidermal growth factor-induced capacitation in rams through suppression of the c-Jun N-terminal kinases (JNKs) mitogen-activated protein kinase (MAPK) pathway [30], while in mice, MT1-mediated cyclic AMP-protein kinase A (cAMP/PKA) signaling regulates capacitation in a concentration-dependent manner [31].

Seasonal fluctuations in seminal plasma MLT concentrations have been reported in rams [6], where elevated MLT levels mitigate oxidative stress by reducing reactive oxygen species (ROS) and nitrogen species in spermatozoa. In contrast, goats exhibit relatively stable MLT concentrations throughout the year; instead, seasonal variations occur in the relative expression of MLT receptors, with higher expression detected during the breeding season. This pattern suggests that sperm quality in goats is influenced more by receptor expression dynamics than by MLT concentration alone [24].

The addition of MLT to cryopreservation media has been shown to enhance sperm quality parameters, including motility, viability, morphology, plasma membrane integrity, and mitochondrial function, in several species [32–35]. MLT may also modulate capacitation status through MT2 receptors: Inhibition of MT2 increases the proportion of capacitated sperm, whereas activation maintains a non-capacitated state under capacitating conditions [33]. However, few studies have evaluated MLT’s role in the freezability of goat sperm, and available data remain inconclusive [36].

Although MLT has been extensively studied for its antioxidant and reproductive regulatory roles in mammals, the mechanistic link between MLT receptor expression and sperm cryotolerance remains poorly understood in small ruminants, particularly goats. Existing studies in rams and other seasonal breeders demonstrate that MLT modulates sperm physiology by influencing capacitation, acrosome reaction, and apoptosis through receptor-mediated pathways such as MT1/cAMP/PKA and MT2/JNK-MAPK signaling. However, species-specific variations in receptor expression and localization imply that the MLT-mediated regulation of sperm function may differ substantially among ruminants.

In goats, MLT supplementation during cryopreservation has yielded inconsistent and inconclusive outcomes, with some studies reporting improvements in motility and membrane integrity, while others found minimal or no benefit. This inconsistency may arise because previous investigations largely focused on exogenous MLT supplementation, overlooking the endogenous MLT system, particularly MT1 and MT2 receptor expression on the sperm plasma membrane and its seasonal dynamics. Furthermore, quantitative correlations between receptor expression, seminal plasma MLT concentration, and post-thaw sperm quality have not been systematically explored in the caprine species. Consequently, the biological significance of MLT receptor machinery in determining sperm freezability and cryosurvival capacity remains uncertain.

Addressing this gap is crucial, as MLT signaling may underlie the natural variation in semen cryoresistance across breeding seasons, potentially explaining why semen collected during the reproductive season exhibits better post-thaw quality. Understanding this relationship could provide new molecular targets for improving cryopreservation protocols and optimizing assisted reproductive technologies in goats.

This study aimed to determine the association between MLT receptor expression and sperm freezability in Saanen goat bucks. Specifically, the objectives were to:

1. Quantify the relative expression levels of MT1 and MT2 receptors in the sperm plasma membrane across seasons using Western blot analysis.
2. Measure the endogenous MLT concentration in seminal plasma by enzyme-linked immunosorbent assay (ELISA).
3. Evaluate post-thaw sperm quality parameters, including motility, viability, membrane integrity, and capacitation status and integrate them into a newly developed Sperm Quality Index (SQI) to assess overall cryosurvival.
4. Analyze the correlations between MLT receptor expression, seminal plasma MLT concentration, and sperm freezability indices to identify potential molecular markers predictive of cryotolerance.

By elucidating how MLT receptor expression influences sperm resilience during freezing and thawing, this research provides novel mechanistic insight into the endogenous MLT system in goats. The findings could guide the development of receptor-targeted or photoperiod-based reproductive management strategies, improving semen cryopreservation outcomes and genetic dissemination efficiency in caprine breeding programs.

## MATERIALS AND METHODS

### Ethical approval

This study was conducted at the Multidisciplinary Research Unit, Laboratory of Animal Reproduction (L2-UIM), Faculty of Superior Studies Cuautitlan, National Autonomous University of Mexico (UNAM), Mexico. All experimental procedures involving animals were approved by the Institutional Subcommittee for the Care of Animals in Experimentation, UNAM (Subcomité Institucional para el Cuidado de Animales en Experimentación, Universidad Nacional Autónoma de México; Approval No. SICUAE.DC-2020/3-3).

### Study period and location

The study was conducted from April 2021 to August 2023 at Facultad de Estudios Superiores Cuautitlán, Universidad Nacional Autónoma de México, Cuautitlán Izcalli 54714, Estado de México, México.

### Animals and management

Seven clinically healthy Saanen bucks (aged 2–3 years; body condition score = 2.5 on a 1–5 scale) were used in this study. All animals were housed individually under standardized management conditions at the Education Center for Agriculture and Animal Breeding (Centro de Enseñanza Agropecuaria), Faculty of Superior Studies Cuautitlan (19° 69´ N, Mexico).

Bucks were maintained under a natural photoperiod (annual variation between the longest and shortest day: 2 h 22 min) and fed a uniform diet consisting of concentrate feed, alfalfa hay, mineral blocks, and water *ad libitum*, with a daily dry matter intake equivalent to 3% of live body weight.

Semen was collected once weekly throughout the year using an artificial vagina. Ejaculates were kept at 37°C until evaluation (~30 min post-collection). A total of 124 ejaculates were obtained (4–5 samples per male per season): 28 in spring, 30 in summer, 32 in autumn, and 34 in winter. Only ejaculates meeting the following criteria were included: progressive motility ≥70%, abnormal sperm <20%, and sperm concentration ≥2.5 × 10^9^ sperm/mL.

### Experimental design

The experimental workflow involved semen evaluation before and after cryopreservation, followed by determination of MLT receptor expression (MT1 and MT2) and MLT concentration in seminal plasma, and correlation analyses with sperm freezability indices.

### Materials and reagents

Microscopic analyses were performed using a Leica DM1000 LED (differential interference contrast) and a Leica DMLS (epifluorescence) microscope (Germany). Protein visualization was carried out using the Odyssey Blot Scanner (LI-COR Imaging System, USA).

All reagents were sourced from Sigma-Aldrich (Germany). The antibodies used for Western blotting were MTNR1A rabbit polyclonal antibody (USBiological, USA), MTNR1B rabbit polyclonal antibody (OriGene, USA), and Alexa Fluor 680 donkey anti-rabbit secondary antibody (Invitrogen, USA). Kaleidoscope molecular weight markers (Bio-Rad, USA) were used. MLT concentrations were quantified using a commercial ELISA kit (MyBioSource, USA).

### Semen evaluation and processing

Immediately after collection, the ejaculates were evaluated macroscopically for volume, color, appearance, and contamination (including blood, hair, and pus). Samples were maintained at 37°C, shielded from light, and transferred to the laboratory within 30 min.

At the laboratory, ejaculates were kept in a 33°C water bath during microscopic assessment. Each sample was diluted 1:100 (v/v) in 0.9% NaCl for microscopic evaluation (except for concentration) and 1:200 (v/v) in 0.3% formalin saline for concentration measurement.

The following sperm parameters were analyzed manually:

- Wave motion and progressive motility (visual assessment)
- Viability (eosin/nigrosine staining)
- Capacitation status (chlortetracycline [CTC] assay)
- Plasma membrane fluidity (merocyanine/MC540 staining)
- Acrosome integrity (phase-contrast microscopy)
- Sperm concentration (Neubauer chamber).

Analyses followed protocols by Martínez-Rodríguez *et al*. [32] and Cardenas-Padilla *et al*. [24].

Aliquots (100 μL) of fresh semen were frozen at –20°C for assessing MLT receptor expression, while seminal plasma-free sperm samples (100 μL) were obtained by double centrifugation to determine MLT concentration.

### Sperm cooling and freezing protocol

A Tris-based freezing medium (250 mM Tris, 28 mM glucose, 104 mM citric acid, 0.05% streptomycin, 500 U/mL penicillin, 12% fresh egg yolk) was used [37], following a two-step dilution and equilibration protocol was applied as described by Cardenas-Padilla *et al*. [24]:

1. Addition of Tris medium without glycerol (10 min equilibration), followed by
2. Addition of Tris medium with 8% glycerol (10 min equilibration).

This achieved a final concentration of 200 × 10^6^ sperm/mL and 4% glycerol. Diluted semen was loaded into 0.25 mL plastic straws, sealed with polyvinyl alcohol, and cooled from 24°C to 5°C over ~3.5 h using a custom cooling receptacle [30] that maintained a rate of 0.088°C/min.

After equilibration, straws were exposed to liquid nitrogen vapors (4 cm above surface) for 15 min, then plunged into liquid nitrogen (−196°C) for storage.

### Thawing and post-thaw evaluation

Thawing was performed by immersing straws (three per ejaculate) in a 37°C water bath for 30 s. Semen was then transferred to dry tubes at the same temperature. Thawed samples were pooled; diluted 1:3 (v/v) in 0.9% NaCl; and evaluated for progressive motility, viability, capacitation status, membrane fluidity, and acrosome integrity as described by Cardenas-Padilla *et al*. [24].

#### Development of the SQI

Data from frozen-thawed sperm were analyzed using a self-developed SQI integrating multiple sperm variables:


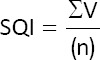


Where V = each sperm variable (%) and n = number of variables.

Two separate indices were established:

- SQI-1: For variables decreasing after freeze-thawing (progressive motility, viability, CTC-F pattern, acrosome integrity).
- SQI-2: For variables increasing after freeze-thawing (CTC-AR pattern, hyper-fluid membranes).

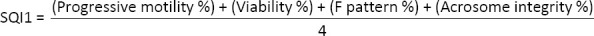



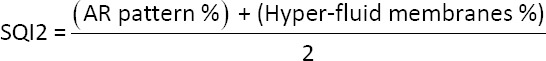


Each parameter was assigned equal weighting, as all assessed traits influence fertilization potential in frozen-thawed sperm [38].

### Expression analysis of MLT receptors (MT1 and MT2)

For receptor analysis, four pooled semen samples (300 μL each), one per season, were prepared from all seven bucks. Western blotting was performed in triplicate following Cárdenas-Padilla *et al*. [24]: (i) Protein extraction, (ii) quantification (spectrophotometry), (iii) electrophoresis, (iv) transfer to membrane, (v) blocking and antibody incubation, and (vi) visualization using the Odyssey Blot Scanner.

Goat retinas served as a positive control for MLT receptor expression, while negative controls omitted the primary antibody. β-actin was used as the loading control for normalization. Band intensities were quantified by densitometry in ImageJ software.

### MLT concentration in seminal plasma

Endogenous MLT levels in seminal plasma were measured in triplicate using a commercial ELISA kit according to manufacturer instructions [24]. The assay sensitivity was 1.0 pg/mL, with intra- and inter-assay coefficients of variation of 4.5% and 8.0%, respectively.

### Statistical analysis

All percentage data (progressive motility, viability, capacitation patterns, membrane fluidity, and acrosome integrity) were arcsine-transformed to normalize distributions. Normality was verified using the Kolmogorov–Smirnov test. Seasonal differences were analyzed using the general linear model in the Statistical Package for the Social Sciences (SPSS) v20.0 (SPSS Inc., Chicago, IL, USA), with significance at p < 0.05. Tukey’s *post hoc* test was applied for multiple comparisons. Associations between MLT receptor expression, MLT concentration, and sperm quality parameters were evaluated using Pearson’s correlation and coefficients of determination (R^2^). Correlation matrices were visualized using the CorrPlot package in R-Studio.

## RESULTS

### Sperm quality indices after cryopreservation

Data on sperm quality parameters after freeze-thawing have been reported by Cardenas-Padilla *et al*. [24] and are not presented here in detail. However, those values were used to calculate the sperm quality indices (SQI-1 and SQI-2) summarized in Table 1.

**Table 1:** SQIs throughout the year in goat bucks.

SQI	Season			

	Spring	Summer	Autumn	Winter
SQI-1	37.15 ± 1.81^a^	36.46 ± 1.40^a^	32.62 ± 1.58^a^	32.81 ± 1.35^a^
SQI-2	70.60 ± 1.67^a^	65.49 ± 1.79^ab^	64.06 ± 1.62^b^	65.35 ± 1.48^ab^

Different letters within the same row differ significantly (p < 0.05). SQI = Sperm quality index.

Significant differences (p < 0.05) were observed in SQI-2 between spring and autumn, corresponding to variables that increased after cryopreservation (e.g., acrosome-reacted [AR] sperm and membrane hyperfluidity). No significant differences were detected between these and other seasons. Conversely, SQI-1, which represents variables that decreased after freezing and thawing (motility, viability, capacitation F pattern, and acrosome integrity), showed no significant seasonal variation.

These results indicate that sperm physiological traits related to capacitation and membrane fluidity are more sensitive to seasonal influence than morphological traits associated with sperm integrity.

### Expression patterns of MLT receptors (MT1 and MT2)

Western blot analysis revealed one distinct band for MT1 (16 kDa) and four bands for MT2 (75, 42, 35, and 28 kDa) in spermatozoa across all seasons. These receptor expression patterns were variable throughout the year.

The MLT concentration in seminal plasma remained statistically unchanged between seasons (Table 2). These findings corroborate previously published observations in goats [24].

**Table 2:** Relative expression of MLT receptors on spermatozoa and MLT concentration in seminal plasma throughout the year in goats.

MLT receptor (MW)	Relative expression of MLT receptors by season			

	Spring	Summer	Autumn	Winter
MT1 (16 kDa)	29.88 ± 2.79^ab^	7.10 ± 1.57^a^	12.55 ± 0.55^ab^	58.49 ± 1.73^b^
MT2 (28 kDa)	10.40 ± 0.44^ab^	3.69 ± 0.41^a^	10.05 ± 0.39^ab^	35.32 ± 0.90^b^
MT2 (35 kDa)	12.89 ± 1.42^ab^	3.17 ± 0.39^a^	5.19 ± 0.44^ab^	14.09 ± 1.49^b^
MT2 (42 kDa)	10.12 ± 1.01^ab^	3.87 ± 1.19^a^	10.44 ± 1.95^ab^	18.18 ± 2.61^b^
MT2 (75 kDa)	15.98 ± 0.78	18.79 ± 0.86	16.09 ± 1.81	14.24 ± 1.98
Concentration of MLT in seminal plasma				
MLT (pg/mL)	334.8 ± 19.14	378.2 ± 17.04	343.6 ± 5.75	366.1 ± 6.11

Values are expressed as mean ± standard error of the mean. Different letters within the same row differ significantly (p < 0.05). MLT = Melatonin.

### Association between MLT receptor expression and sperm quality variables

Correlation analyses were conducted to determine the relationship between MLT receptor expression, MLT concentration, and sperm quality parameters after cryopreservation.

The expression levels of both MT1 and MT2 receptors showed weak to moderate associations with most sperm parameters, including motility, viability, capacitated sperm with intact acrosomes (CTC-B pattern), membrane fluidity, and acrosome integrity (R^2^ < 0.6).

**Figure 1:**
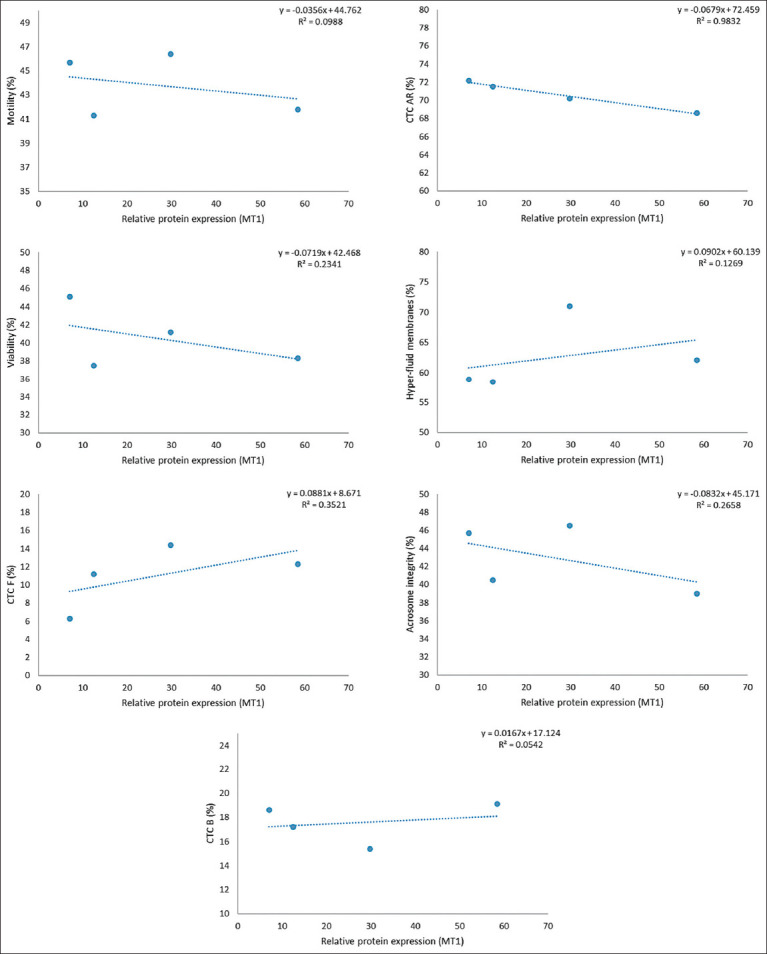
Association between each variable evaluated in frozen-thawed sperm and the relative expression of the MT1 receptor (16 kDa) in goats’ spermatozoa throughout the year.

**Figure 2:**
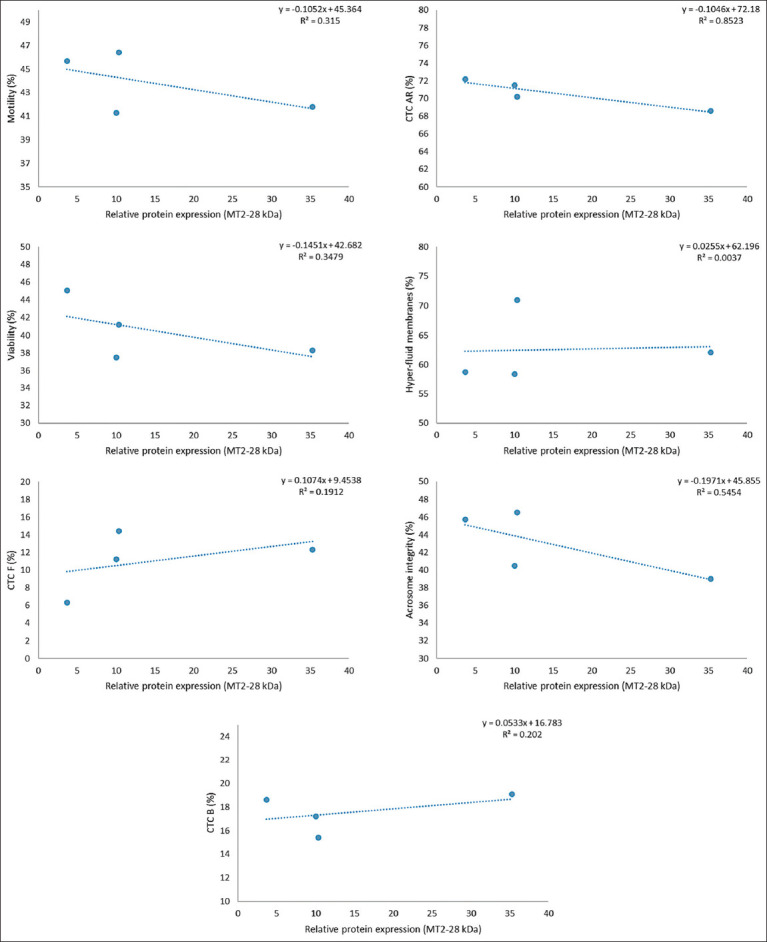
Association between each variable evaluated in frozen-thawed sperm and the relative expression of the MT2 receptor (MW = 28 kDa) in goats’ spermatozoa throughout the year.

**Figure 3:**
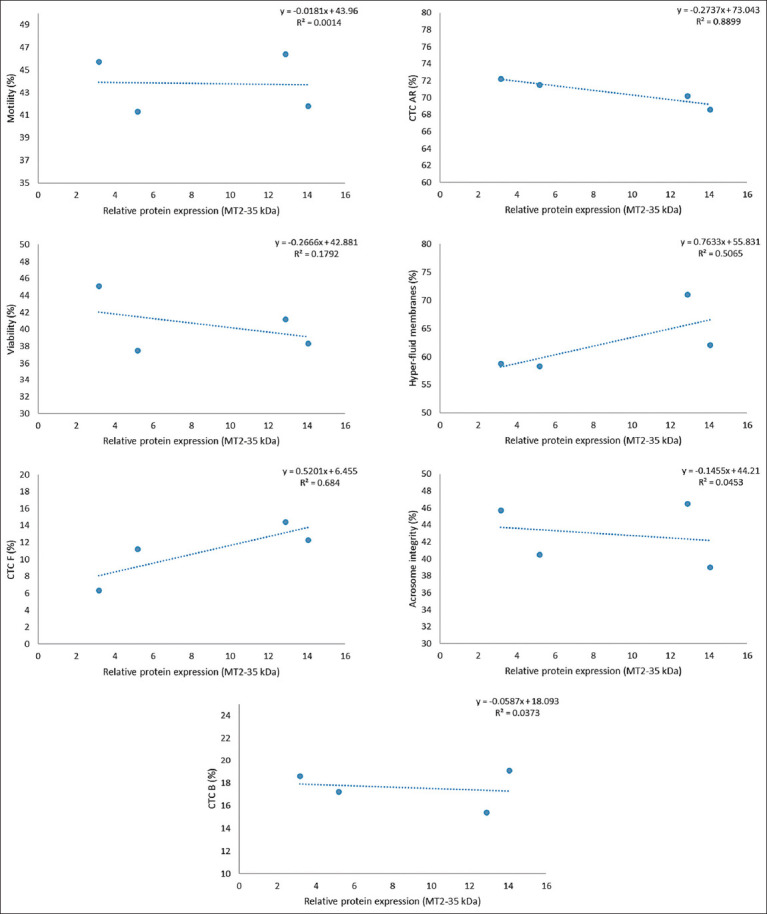
Association between each variable evaluated in frozen-thawed sperm and relative expression of MT2 receptor (MW = 35 kDa) in goats’ spermatozoa throughout the year.

**Figure 4:**
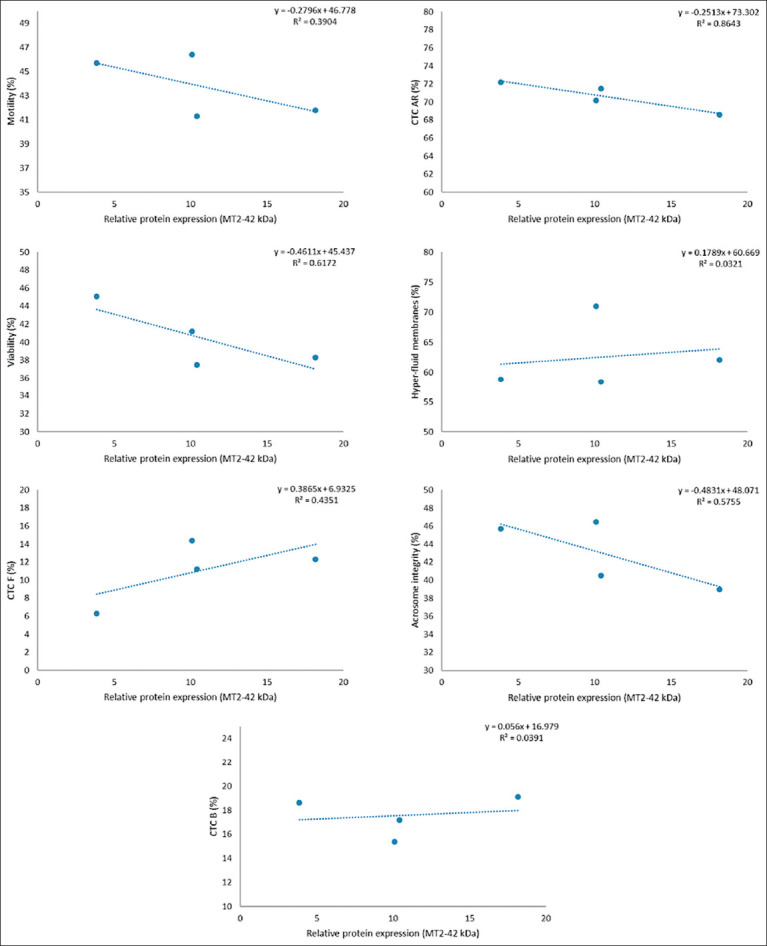
Association between each variable evaluated in frozen-thawed sperm and relative expression of MT2 receptor (MW = 42 kDa) in goats’ spermatozoa throughout the year.

**Figure 5:**
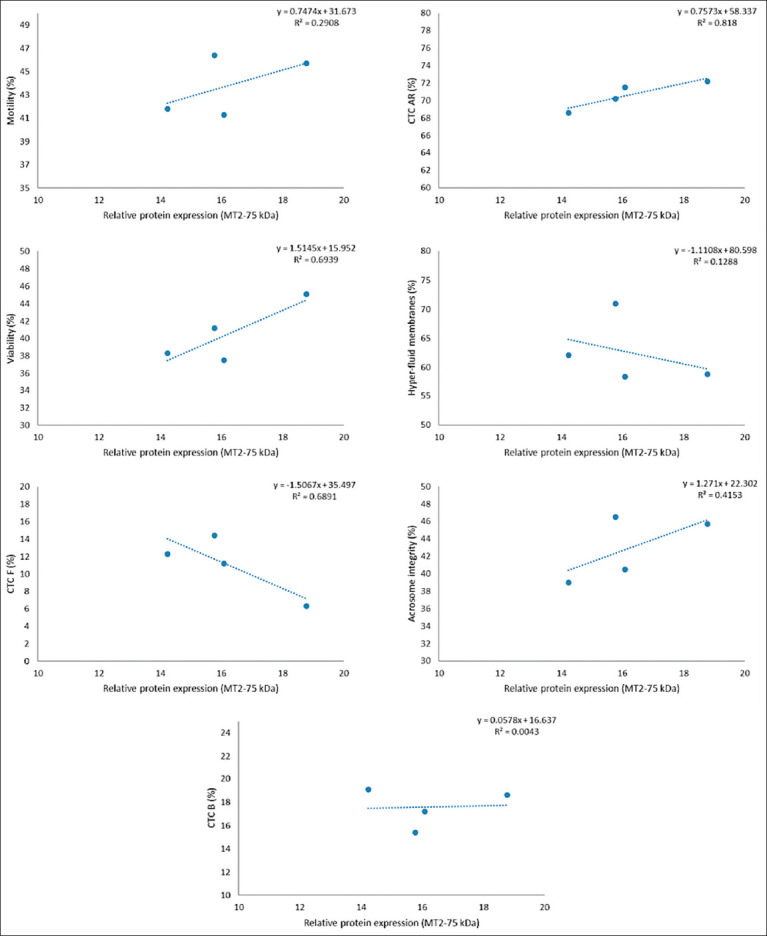
Association between each variable evaluated in frozen-thawed sperm and relative expression of MT2 receptor (MW = 75 kDa) in goats´ spermatozoa throughout the year.

**Figure 6:**
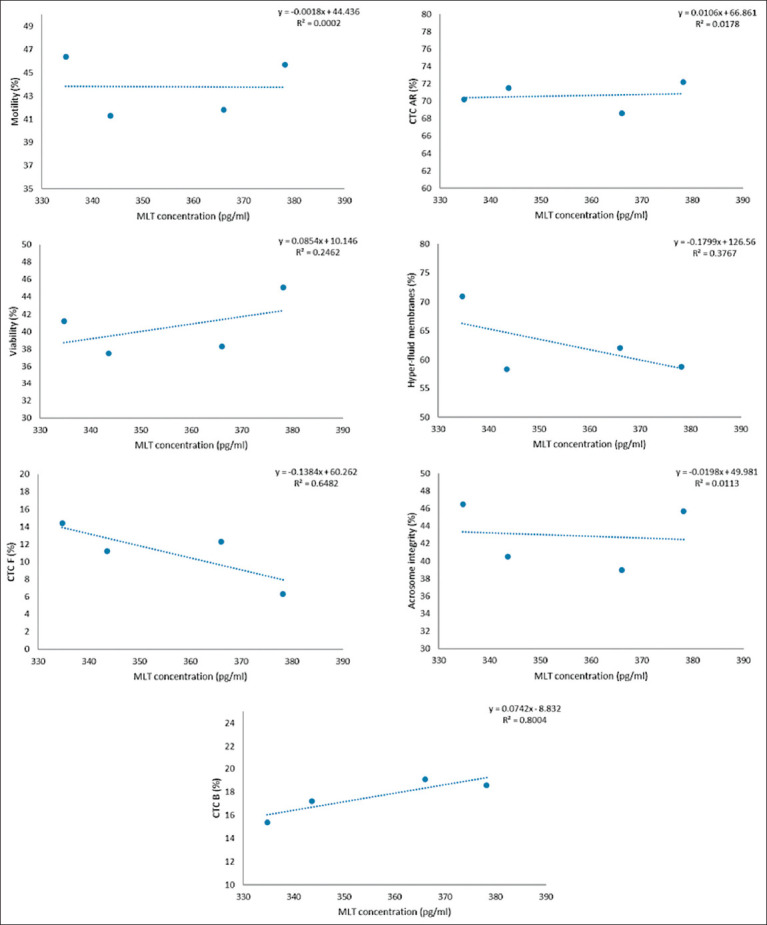
Association between each variable evaluated in frozen-thawed sperm and MLT concentration in the seminal plasma of goats throughout the year. MLT = Melatonin.

However, a strong inverse relationship (R^2^ ≥ 0.8) was observed between receptor expression and the percentage of AR spermatozoa (CTC-AR pattern), as illustrated in Figures 1-6. This indicates that higher MT1 and MT2 receptor expression correlates with reduced incidence of premature acrosome reactions following freeze-thawing.

### Association between MLT concentration and sperm capacitation

The MLT concentration in seminal plasma was positively correlated with the proportion of capacitated spermatozoa with intact acrosomes (CTC-B pattern), showing a high coefficient of determination (R^2^ ≥ 0.8). This suggests that MLT may help maintain membrane stability and delay premature capacitation during cryopreservation.

### Correlation matrix analysis

**Figure 7:**
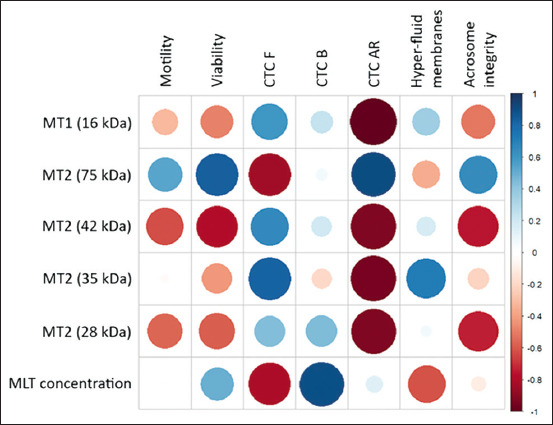
CorrPlot obtained in RStudio for each variable and its association with the expression of MLT receptors and MLT concentration in seminal plasma throughout the year. The circles show the absolute values of the corresponding determination coefficients. Positive coefficients are shown in blue while negative coefficients are shown in red. The color intensity is proportional to the correlation coefficients. MLT = Melatonin.

A comprehensive correlation matrix was generated using the CorrPlot package in RStudio (Posit Software, PBC, Boston, MA, USA) to visualize the relationships among sperm quality traits (motility, viability, capacitation patterns, membrane fluidity, and acrosome integrity), MLT receptor expression, and MLT concentration in seminal plasma across seasons (Figure 7).

The graphical representation highlighted both positive (blue) and negative (red) associations, with color intensity corresponding to the magnitude of the correlation coefficients (R and R^2^). Notably, the strongest associations were observed between MT receptor expression and CTC-AR pattern, confirming a direct mechanistic relationship between receptor-mediated MLT signaling and sperm cryotolerance.

## DISCUSSION

### Development and relevance of the SQI

In this study, two comprehensive indices of sperm quality were developed to evaluate the impact of cryopreservation on goat semen. Unlike most conventional assessments that emphasize only motility and morphology as fertility predictors [38], the proposed SQI integrates multiple parameters representing both structural and functional sperm attributes. Each variable was assigned equal weight, as every component contributes to the fertilization potential of cryopreserved spermatozoa.

Notably, SQI-2, representing variables that increase following cryopreservation (e.g., acrosome reaction and membrane hyperfluidity), differed significantly (p < 0.05) between spring and autumn. This seasonal variation suggests that physiological parameters, such as capacitation status and plasma membrane fluidity, are more susceptible to cryogenic stress than morphological parameters, such as acrosome integrity. Therefore, SQI-2 may serve as a sensitive indicator for detecting subtle physiological disruptions in frozen-thawed sperm.

### Relationship between MLT receptor expression and sperm function

A strong inverse correlation (R^2^ ≥ 0.8) was observed between AR spermatozoa (CTC-AR pattern) and the expression levels of MT1 and MT2 receptors in sperm plasma membranes across seasons. The correlation coefficient exceeded 0.9 for all receptor bands, indicating that higher receptor expression is associated with a reduced proportion of AR spermatozoa.

This finding suggests that elevated MLT receptor expression during the reproductive season may mitigate cryocapacitation, a process characterized by premature capacitation and the acrosome reaction triggered by cryogenic stress [39]. Consequently, cryopreservation performed during the breeding season may yield sperm with improved acrosomal stability and a lower risk of cryodamage.

### Role of MLT and its receptors in capacitation modulation

The positive correlation between MLT concentration in seminal plasma and the proportion of capacitated spermatozoa with intact acrosomes (CTC-B pattern) suggests that MLT actively modulates the dynamics of capacitation. Similar receptor-mediated effects have been reported in other species, where MLT regulates sperm function through the MT1/cAMP/PKA and MT2/JNK-MAPK signaling pathways [30, 31].

Although the precise biochemical pathway in goats remains to be elucidated, the evidence indicates that MLT may act through two complementary mechanisms:

1. Antioxidant protection by scavenging ROS and stabilizing membranes.
2. Receptor-mediated signaling by modulating intracellular calcium influx and cholesterol efflux, which are key regulators of capacitation.

### MLT’s role in cryotolerance and seasonal adaptation

MLT is a potent free radical scavenger (indolamine) known to enhance sperm cryotolerance by reducing oxidative damage during freezing and thawing [10, 36]. In rams, MLT exerts its protective effect primarily through MT2 receptor activation, influencing capacitation and apoptosis [40].

While seasonal variations in seminal MLT concentration are well documented in rams [6], no such variation was detected in goats. However, receptor expression varied significantly across seasons, suggesting that MLT responsiveness in goats depends more on receptor density than on circulating MLT concentration. This receptor-driven modulation could explain why goats exhibit consistently high MLT levels but still show seasonal changes in sperm freezability.

Casao *et al*. [41] proposed that MLT concentration directly influences capacitation both during cryopreservation and post-mating. Interestingly, the baseline MLT levels in goats are reportedly higher than in other species, implying a stronger decapacitating effect of MLT in goats. Our findings support this hypothesis, demonstrating that enhanced expression of the MT1 and MT2 receptors correlates with improved sperm stability and freezability, even in the absence of significant fluctuations in MLT levels.

### Comparative perspective and implications for reproductive biotechnology

A recent study by Doghbri *et al*. [23] on camels have shown seasonal increases in seminal plasma MLT during the breeding period, along with the detection of MT1 and MT2 receptors on sperm membranes. Although these findings suggest a role for MLT in enhancing sperm quality, the dynamics of receptor expression were not evaluated. The present study extends this understanding by demonstrating that receptor expression itself, rather than MLT concentration alone, plays a decisive role in maintaining sperm function under cryogenic stress.

These insights highlight the potential of MLT receptor profiling as a biomarker for sperm cryo-resilience and suggest that seasonal modulation of MLT signaling could be strategically leveraged to enhance the outcomes of artificial insemination and semen cryobanking programs in goats and other small ruminants.

## CONCLUSION

This study demonstrated that sperm freezability in Saanen goat bucks is closely associated with both MLT receptor expression and MLT concentration in seminal plasma. Western blot analysis identified the presence of MT1 (16 kDa) and MT2 (75, 42, 35, and 28 kDa) receptors on the sperm plasma membrane across all seasons. Although the overall MLT concentration in seminal plasma remained constant throughout the year, seasonal variation in receptor expression was evident. A strong negative correlation (R > −0.9; R^2^ > 0.8) was found between receptor expression (MT1 and MT2) and the proportion of AR spermatozoa (CTC-AR pattern), indicating that higher receptor expression reduces premature acrosomal loss during cryopreservation. Conversely, a strong positive correlation (R > 0.9; R^2^ > 0.8) was observed between seminal MLT concentration and capacitated sperm with intact acrosomes (CTC-B pattern), suggesting that MLT contributes to membrane stabilization and controlled capacitation. The newly developed Sperm Quality Indices (SQI-1 and SQI-2) further revealed that physiological sperm parameters such as capacitation and membrane fluidity are more sensitive to cryogenic stress than morphological traits like acrosome integrity.

These findings provide a molecular explanation for the seasonal variation in semen freezability in goats. Enhancing MLT receptor expression or optimizing MLT signaling could improve post-thaw sperm survival and fertilizing ability. The results indicate that semen collection during the breeding season, when receptor expression is naturally elevated, may enhance the success of artificial insemination and cryobanking programs. Moreover, incorporating MLT-based antioxidants or receptor-targeted treatments into freezing extenders could minimize cryocapacitation and preserve sperm functionality after thawing.

This work is strengthened by its comprehensive integration of biochemical, physiological, and cryobiological data using a novel SQI, which offers a quantitative framework for assessing cryosurvival. The study also covered a complete annual cycle, ensuring robust seasonal representation. However, it was limited to a single breed (Saanen goats) and controlled environmental conditions, and *in vivo* fertility validation was not conducted. In addition, the molecular pathways linking MLT receptor activation to sperm capacitation, such as cAMP/PKA or JNK-MAPK signaling, were not experimentally confirmed.

Future research should focus on elucidating the intracellular signaling mechanisms underlying MT1 and MT2 receptor activity, developing receptor modulation strategies through photoperiod management or dietary MLT supplementation, and conducting comparative studies across breeds and ruminant species. Field-level validation of SQI and receptor expression as predictors of fertility will also be essential.

In conclusion, MLT receptor expression, rather than MLT concentration alone, plays a decisive role in determining sperm cryotolerance in goats. By maintaining acrosomal integrity and regulating capacitation, the MLT-receptor system enhances sperm resilience to freezing stress. These findings provide new molecular insight into the role of endogenous MLT in male fertility and open opportunities to design receptor-guided cryopreservation strategies that improve reproductive efficiency and genetic conservation in caprine species.

## DATA AVAILABILITY

The supplementary data can be made available from the corresponding author upon request.

## AUTHORS’ CONTRIBUTIONS

AJCP: Conducted the laboratory experiments and drafted the manuscript. AJCP and AM: Conceptualized, designed, and supervised the study, performed statistical analysis, and revised and edited the manuscript. AM: Project administration. All authors have read and approved the final version of the manuscript.
